# Expression Profile of miRs in Mesial Temporal Lobe Epilepsy: Systematic Review

**DOI:** 10.3390/ijms23020951

**Published:** 2022-01-16

**Authors:** Kristina D. Yakovleva, Diana V. Dmitrenko, Iulia S. Panina, Anna A. Usoltseva, Kirill A. Gazenkampf, Olga V. Konovalenko, Elena A. Kantimirova, Maxim A. Novitsky, Regina F. Nasyrova, Natalia A. Shnayder

**Affiliations:** 1 Department of Medical Genetics and Clinical Neurophysiology, Institute of Postgraduate Education, V.F. Voino-Yasenetsky Krasnoyarsk State Medical University, 660022 Krasnoyarsk, Russia; kris_995@mail.ru (K.D.Y.); mrs.yuliapanina@mail.ru (I.S.P.); a.usoltseva@list.ru (A.A.U.); hassenkampf@mail.ru (K.A.G.); konovalenko-olga@inbox.ru (O.V.K.); kantilea@mail.ru (E.A.K.); 2 Institute of Personalized Psychiatry and Neurology, Shared Core Facilities, V.M. Bekhterev National Medical Research Center for Psychiatry and Neurology, 192019 Saint Petersburg, Russia; maximnovitsky93@gmail.com (M.A.N.); nreginaf77@gmail.com (R.F.N.); 3 International Centre for Education and Research in Neuropsychiatry, Samara State Medical University, 443099 Samara, Russia; 4 Shared Core Facilities “Molecular and Cell Technologies”, V.F. Voino-Yasenetsky Krasnoyarsk State Medical University, 660022 Krasnoyarsk, Russia

**Keywords:** biomarker, diagnostic panel, genetics, epilepsy, epileptogenesis, microRNA, seizure, temporal lobe epilepsy, therapeutic resistance

## Abstract

Temporal lobe epilepsy (TLE) is one of the most common forms of focal epilepsy in children and adults. TLE is characterized by variable onset and seizures. Moreover, this form of epilepsy is often resistant to pharmacotherapy. The search for new mechanisms for the development of TLE may provide us with a key to the development of new diagnostic methods and a personalized approach to the treatment. In recent years, the role of non-coding ribonucleic acids (RNA) has been actively studied, among which microRNA (miR) is of the greatest interest. (1) Background: The purpose of the systematic review is to analyze the studies carried out on the role of miRs in the development of mesial TLE (mTLE) and update the existing knowledge about the biomarkers of this disease. (2) Methods: The search for publications was carried out in the databases PubMed, Springer, Web of Science, Clinicalkeys, Scopus, OxfordPress, Cochrane. The search was carried out using keywords and combinations. We analyzed publications for 2016–2021, including original studies in an animal model of TLE and with the participation of patients with TLE, thematic and systemic reviews, and Cochrane reviews. (3) Results: this thematic review showed that miR‒155, miR‒153, miR‒361‒5p, miR‒4668‒5p, miR‒8071, miR‒197‒5p, miR‒145, miR‒181, miR‒199a, miR‒1183, miR‒129‒2‒3p, miR‒143‒3p (upregulation), miR–134, miR‒0067835, and miR‒153 (downregulation) can be considered as biomarkers of mTLE. However, the roles of miR‒146a, miR‒142, miR‒106b, and miR‒223 are questionable and need further study. (4) Conclusion: In the future, it will be possible to consider previously studied miRs, which have high specificity and sensitivity in mTLE, as prognostic biomarkers (predictors) of the risk of developing this disease in patients with potentially epileptogenic structural damage to the mesial regions of the temporal lobe of the brain (congenital disorders of the neuronal migration and neurogenesis, brain injury, neuro-inflammation, tumor, impaired blood supply, neurodegeneration, etc.).

## 1. Introduction

Temporal lobe epilepsy (TLE) is one of the most common and heterogeneous forms of focal epilepsy in children [1] and adults [2]. TLE is characterized by a variable onset [3] and different epileptic seizures [4]. Furthermore, this form of epilepsy is often resistant to pharmacotherapy [5,6,7]. The etiology of TLE is variable and includes the influence of external environmental and genetic factors: genetic [8] and epigenetic mechanisms [9]; family forms TLE [10,11]; chronic neuro-inflammation [12]; neuronal death/apoptosis [13]; disorders of neurogenesis [9] (hippocampal sclerosis [14], focal cortical dysplasia [15,16], polymicrogyria, and nodal heterotopy [17]).

There are some forms of TLE, which are: neocortical TLE, mesial TLE without hippocampal sclerosis (mTLE without HS), and mesial TLE with hippocampal sclerosis (mTLE-HS). Neocortical TLE is more common in young children, but mTLE is most common and occurs in adolescents and adults [4,18]. mTLE, including the familial form, was first described as an epileptic syndrome with persistent mental and autonomic seizures that are not associated with hippocampal sclerosis or febrile seizures [4]. The genetics of this condition is understudied, although several candidate genes have been identified as responsible for familial mTLE and multifactorial mTLE [19]. The role of epigenetic mechanisms and circulating microRNAs (miRs) in the development of mTLE is important for the development of personalized approaches to the diagnosis and treatment of TLE; unfortunately, it is insufficiently studied [20]. The search for new mechanisms for the development of mTLE may provide us with a key to the elaboration of new diagnostic methods and personalized therapies.

Evidence suggests that ribonucleic acid (RNA) is not only functional as a messenger between DNA and protein but also involved in the regulation of genome organization and gene expression, which is increasingly elaborate in complex organisms. Regulatory RNA seems to operate at many levels; in particular, it plays an important part in the epigenetic processes that control differentiation and development. These discoveries suggest a central role for RNA in human evolution and ontogeny [21]. In recent years, the role of regulatory (noncoding) RNA in epileptogenesis has been actively studied.

Non-coding RNAs are a large family of RNAs that are not coding for known proteins. In general, non-coding RNAs can be classified according to their length into small (<200 nucleotides) and long (>200 nucleotides) RNAs or according to their function as housekeeping and regulatory RNAs [22]. About 17 categories of non-coding RNA molecules have been identified so far; among them, transfer RNAs, ribosomal RNAs, small nucleolar RNAs, endogenous small interfering RNAs, sno-derived RNAs, transcription initiation RNAs, microRNA-offset-RNAs, circular RNAs, vault RNAs, microRNAs (miRNAs), small interfering RNAs (siRNAs), small nuclear RNAs, extracellular RNAs, piwi-interacting RNAs, small Cajal body RNAs, long intergenic non-coding RNAs, and long non-coding RNAs (lncRNAs) are known [23]. They regulate transcription, influence translation of coding genes, are components of the protein synthesis machinery, regulate each other, e.g., modify ribosomal RNAs, and lncRNAs can counteract miRNAs by sequestering them (miRNA sponges) [24]. Moreover, many physiological processes are regulated by non-coding RNAs, including development, gametogenesis, stress, immune response, tumourogenesis, and epileptogenesis [23].

miRNAs are evolutionarily conserved, small noncoding RNAs found in most plants and animals [25]. It is known that miR is an RNA about 18–24 nucleotides long, it belongs to the class of small non-coding RNAs, and it plays a crucial role in the post-transcriptional regulation of gene expression, cellular metabolic pathways, and developmental events [26]. The human genome includes 1917 annotated hairpin and 2654 mature miRs [27].

Previously, the role and importance of miRs was unknown; miR was even referred to as so-called “genetic junk”, and the research focused on deoxyribonucleic acid (DNA). The classic dogma that DNA is transcribed into RNA, which is then translated into protein, has postponed the study of all non-protein coding and non-coding sequences for many decades. Since the 1990s, the attention of researchers has been attracted by miRs, but they are still actively studied [28]. miRNAs function post-transcriptionally by usually base-pairing to the mRNA 3’-untranslated regions to repress protein synthesis by mechanisms that are not fully understood [29]. However, many, if not most, protein-coding transcripts are targets for miRNA regulation [30,31], for which miRNAs can, in some cases, regulate large numbers of target mRNAs [29], and reciprocally, many mRNAs contain target sites for many miRNAs [32], although the implied regulatory logic of this complex multiplex arrangement has not been explained. The targets of miRNAs are usually thought to be mRNAs but may also include other RNAs [33]. The miRNA pathway regulates post-transcriptional gene expression through the deadenylation and translation repression of target mRNAs. miRNA genes, which are about 1% of all human genes, regulate protein production for 10% or more of all human genes [30].

Recent studies revealed that the early step of translation initiation is the target of “pure” translation repression by the miRNA pathway. Moreover, particularly in animals, the miRNA pathway is required for neuronal development, differentiation, and plasticity. In addition, some functions of miRNAs are regulated by RNA-binding proteins (RBPs) in neuronal cells [25]. Biologically, miRNAs have been shown to regulate many physiological, developmental, and disease processes, including, for example, pluripotency [34], epithelial-mesenchymal transition and metastasis [35], neural plasticity, learning, and memory [36,37,38], among others. Recent studies show the involvement of RNA-mediated gene silencing in neurogenesis, neural differentiation, synaptic plasticity, and neurologic and psychiatric diseases [39].

The purpose of the systematic review is to analyze the role of miRs in the development of mTLE and update the existing knowledge about the biomarkers of this disease (on the materials of some recent studies).

## 2. Methods and Materials

A search was carried out for English language publications in the databases PubMed, Springer, Web of Science, Clinicalkeys, Scopus, OxfordPress, and Cochrain. The search was performed with the use of keywords and word combinations: biomarker; genetics; epilepsy; epileptogenesis; microRNA; seizure; temporal lobe epilepsy; therapeutic resistance. We analyzed publications issued from 2016 to 2021, including original studies in an animal model of TLE and with the participation of patients with TLE, thematic and systemic reviews, and Cocrane reviews.

**Figure 1:**
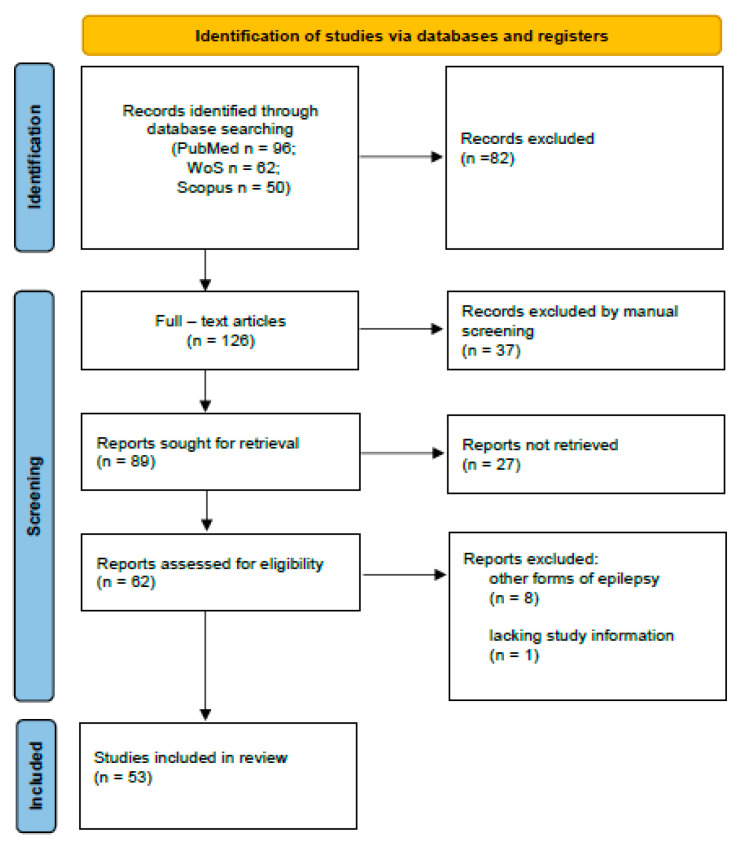
Flow chart diagram visualizing the database searches, number of publications identified, screened, and final full texts included in the present systematic review.

The systematic review was carried out according to the Preferred Reporting Items for Systematic Review and Meta-Analysis (PRISMA 2020). A flow chart is provided in Figure 1.

In total, we analyzed 96 publications. The studies were carried out with the use of biological fluids (serum, cerebrospinal fluid), including the exosomes of blood plasma and brain tissue, to identify circulating miRs as biomarkers of epileptogenesis or predictors of the development of epileptic seizures in TLE and therapeutic resistance. Moreover, earlier publications of historical interest have been included in this review.

## 3. Results

The main studies of miRs in TLE are aimed at finding biomarkers associated with epileptogenesis and the development of epileptic seizures [40]. Furthermore, the role of miRs in the diagnosis of TLE in general is being studied [41,42,43], as well as mTLE [44,45], mTLE-HS [46], and therapeutically resistant TLE [47,48]. A high level of miR (AUC > 0.800) as a biomarker of epilepsy has been confirmed by acceptable sensitivity and specificity. However, most of the previous studies were carried out in small cohorts of patients, often not validated. The vast majority of studies do not report threshold values that would distinguish a group of patients with epilepsy from control groups. However, the specificity of miR as a biomarker of epilepsy relative to other brain diseases has not been analyzed, although this is important for the differential diagnosis of epilepsy [49].

Early functional studies linked the influence of miRs on the development of epileptic seizures with neuro-inflammation and microstructural changes in neurons. At the same time, it was shown that the level of pro-inflammatory cytokines increases in epilepsy or after events provoking epilepsy and correlates with an increase in the level of related miRs [50].

The first human TLE miR study was published in 2010 and reported an increase in hippocampal miR–146a levels associated with the control of inflammatory responses [51] and regulation of expression of Toll-like receptors (TLRs) and cytokine signaling pathways [52]. The experimental study by Li et al. [53] found that overexpression of miR‒146a can regulate interleukin 1 beta (IL‒1β) expression and decrease the expression of compliment system factor H (CFH), while miR‒146a hypo-expression can decrease IL‒1β expression and increase CFH expression in the hippocampus in rats with a chronic mesial TLE. The study carried out by Jimenez-Mateos et al. [52] showed that miR‒let‒7b expressed in the brain can activate Toll‒like receptors of type 7 (TLR‒7) and result in neuronal death. It was also shown that IL‒1β regulates the expression of miR‒146a in cultures of human astrocytes [53]. It has been hypothesized that the immune system cannot only be regulated by miR, but it itself influences the expression and activity of miR [54]. It was revealed that, in the normal brain, miR‒146a is expressed by neurons and not by glia. After status epilepticus was diagnosed, hippocampal levels of miR‒146a were increased both in rats of different ages and in the resected hippocampus in adults and children with therapeutically resistant mTLE. Elevated levels of miR‒146a are found in neurons and astrocytes, but not in microglia. This indicates the specificity of the types of brain cells in which this miR is expressed. However, it is not clear whether miR‒146a expression is increased in mTLE patients without HS. The mechanism for increasing miR‒146a levels can be mediated through IL‒1, while tumor necrosis factor alpha (TNFα) does not stimulate miR‒146a expression [51]. Along with miR‒146a, the role of miR‒155 and miR‒21 in the immune response, through modulation of the Toll‒like receptor (TLR), is shown. Both of these miRs are enriched in glial cells, including astrocytes, and are likely to play a role in neuro-inflammatory signaling pathways [55].

Kan et al. [56] showed that the expression of miR‒221 and miR‒222 is suppressed in astrocytes in the hippocampus in patients with mTLE-HS compared with the control group. These miRs reduce the level of type 1 intercellular adhesion molecules (ICAM1), also known as CD54. HS provokes an increase in the expression of ICAM1 by astrocytes. ICAM1 is associated with the recruitment, accumulation, and activation of leukocytes and microglia. These cells express ICAM1 binding partners, such as the heterodimeric integrin of the β2-integrin subfamily (LFA-1) and integrin alpha M beta 2 (Mac-1); as a result of their interaction, the production of inflammatory mediators by astrocytes and immune cells can be triggered. These effects may contribute to enhancing and maintaining the local immune response that is observed in patients with mTLE-HS. For example, dysregulation of miR‒134 altered the number and volume of dendritic spines on excitatory neurons in the mesial regions of the temporal lobe, whereas miR‒146a, miR‒221, and miR‒222 can control immune responses through target molecules, such as IL‒1β and ICAM1 [50,51,56].

Changes in the expression of miRs involved in inflammatory reactions (miR‒146a and miR‒155), neuronal proliferation, differentiation, migration, and organization (miR‒124, miR‒134, miR‒132, and miR‒196b) were observed in the hippocampus of patients with mTLE with HS [51,57,58,59]. Additionally, overexpression of miR‒146a in patients with epilepsy was found to be a risk factor for the formation of therapeutic resistance to antiepileptic drugs (AEDs), especially in carriers of the single nucleotide variant (SNV) rs57095329 of the *MIR146A* gene. Carriage of this SNV predisposes to a decrease in the severity of chronic neuro-inflammation in the central nervous system (CNS) in the latent stage of epilepsy development, since miR‒146a can limit the excessive pro-inflammatory response under physiological conditions [60].

### 3.1. Neurodegeneration

The progressive loss of neurons in vulnerable areas of the hippocampus is one of the pathways underlying epileptogenesis. This pathway contributes to the development of super-excitable neural networks due to unbalanced inhibition of excitation and influence on the synaptic reorganization of intact neurons. Moreover, recurrent seizures in therapeutically resistant mTLE can lead to progressive neuronal loss, predominantly in the CA1 and CA3 regions of the hippocampus, while the CA2 neurons are relatively intact [61]. It has been shown that a number of miRs regulate apoptosis of neurons and the regulation of some processes of apoptosis changes after events provoking epilepsy. Changes in expression of miR‒132 [62], miR‒34 [63], and miR‒124 [64] in functional studies demonstrate an increase or decrease in neuronal loss after status epilepticus has been diagnosed.

miRs dysregulation in TLE with hippocampal sclerosis may be a response to epileptic seizure-induced neuronal death and, therefore, may play a role in the pathogenesis of mTLE. Genomic analysis of DNA methylation in hippocampal tissue in patients with mTLE revealed differences in the methylation state of several miR genes. Inverse correlations between methylation status and miR expression in hippocampal neuron samples were revealed [65,66]. Thus, in mTLE, suppression of miR–15a–5p expression is observed. This can lead to a decrease in its control activity on targets, including the ubiquitin ligase F-Box and WD repeat domain containing 7 (FBXW7), which destabilizes cyclin E, leading to blocking of the cell cycle in the S-phase. As a result, these processes partially reflect the inhibition of neurogenesis, which, with a parallel increase in neuronal apoptosis, leads to their loss observed in patients with TLE [48].

In a study by Kaalund et al. [54], miR‒204 and miR‒218 levels were significantly reduced in tissue samples from the mesial regions of the temporal lobe from patients with mesial TLE with hippocampal sclerosis. Both of these miRs were expressed in neurons in all regions of the hippocampus. Additionally, pronounced changes in the expression of miR‒204 and miR‒218 were found during intrauterine development of the hippocampus in an animal model of TLE [54].

Fang et al. [67] showed that slit guidance ligand 2 (SLIT2) is activated in patients with therapeutically resistant mTLE without HS. SLIT2 expression shifts from predominantly glial to neuronal during epileptogenesis. Another indicator of the involvement of miR‒218 in synaptic plasticity and, possibly, epileptogenesis, is its regulatory effect on the translation of glutamate metabotropic receptor 1 (GRM1). Research has shown the roles of miR‒204 and miR‒218 in the suppression of the messenger RNA (mRNA) *GRM1* gene, which encodes the metabotropic glutamate receptor (mGluR1). However, studies of GRM1 expression in mTLE-HS have had conflicting results [54].

Ashhab et al. [57] suggested that the “TNF–α/miR–155” axis modulates as a new therapeutic target in reducing the rate of neurodegeneration in mesial TLE. The role of TNF-α and miR‒155, which have similar expression patterns at three stages of mesial TLE development, has been shown. Expression of TNF‒α and miR‒155 was significantly increased in acute and chronic stages of mesial TLE in an animal model in rats, as well as in their offspring with mTLE.

### 3.2. Neurogenesis

Status epilepticus and other events that provoke a severe course of TLE can enhance neurogenesis within the sub-granular zone of the hippocampus by activating a sub-population of resting neural stem cells [68]. However, the functional significance of the activation of neurogenesis in the development of TLE is not well understood [69,70,71,72]. At the same time, miRs have been shown to promote activation of neural stem cells after the diagnosis of status epilepticus and cell migration from stem cells. miR‒19 [73], miR‒124 [74,75], and и miR‒132 [76] are of key importance for determining the development of neurons, migration, and integration of neurons [50].

Expression of miR‒124 in HeLa cells shifts the cell transcriptome towards neurons, indicating pro-neuronal activity of miR‒124 [50]. Irreversible loss of miR‒124 function in neural stem cells causes a decrease in neurogenesis and an increase in gliogenesis [74] in the mediobasal regions of the temporal lobes of the brain, which is important for the development of mesial TLE. miR‒124 may play a crucial role in status epilepticus-induced neurogenesis by functioning in combination with miR‒137 [77,78] to control the activity of caspase-3, which regulates mitochondria-dependent pathways of apoptosis in neural progenitor cells [64]. Additionally, miR‒124 is involved in the regulation of neuron restrictive silencing factor (NRSF) [79] as a transcriptional repressor that represses critical neuronal genes in epileptogenesis, including hyperpolarization activated cyclic nucleotide gated potassium channel 1 (*HCN1*) and potassium-chloride transporter member 5 (*KCC2*) [80,81,82]. NRSF is important for maintaining the pool of adult neural stem cells and coordinates their differentiation, which is specific at different stages [83] and is probably achieved through the regulation of miR‒9 and miR‒124 in cyclic feedback [84,85].

miR‒128 controls neurogenesis and synaptogenesis, but its potential role for post-transcriptional mechanisms due to accumulation of miR‒128 in post-mitotic neurons during corticogenesis is not clear. Changes in the expression of miR‒128 can lead to a decrease in the development of dendrites and their branching, which is associated with impaired electrical excitability of neurons [85]. Furthermore, an additional function of miR‒128 has been reported in the study by Lin et al. [86]: overexpression of miR‒128 is associated with the development of the response of fear and can also contribute to the extinction of the learned fear response to previously experienced negative events and is necessary for this, including the suppression of memory caused by the fear of recurrence of epileptic seizures. This may be of interest in epileptology, since the retention of the memory of pre-existing epileptic seizures can lead to the induction of new epileptic seizures, therapeutic resistance to AEDs, and pseudo-resistance of TLE, as well as a decrease in the number of positive outcomes of surgical treatment of therapeutically resistant TLE in early and long-term postoperative periods [87]. However, the role of miR‒128 in the regulation of learning processes is currently unknown. It was shown that hypo-expression or complete suppression of miR‒128‒2 expression in the CNS led to hyperactive motor behavior and severe epileptic seizures in experimental animals. Selective ablation of miR‒128‒2 post-mitotic forebrain neurons led to neuronal hyperactivation and seizures, which could only be stopped by ectopic expression of miR‒128. But the phenotype of the miR‒128 deletion in relation to the development of the cerebral cortex in humans is not known [65].

Franzoni et al. [85] studied the role of miR‒128 in the regulation of migration, growth, and neurogenesis through its effect on the *Phf6* gene, which is involved in the control of the intellectual development of mammals, in an animal model (mice). This is also of interest in TLE, since damage to the hippocampus leads not only to the development of epileptic seizures, but also to impairment of memory consolidation underlying learning and intellectual development. The authors showed that miR‒128 regulates dendrite branching and neuronal excitability. Having analyzed the expression pattern of miR‒128 during neocorticogenesis and synaptogenesis, the authors presented evidence that miR‒128 may be part of the regulatory switch required for the transition from neuronal migration to their growth and functional maturation. Moreover, the authors tested the effect of premature expression of miR‒128 on radial migration of neurons, which is also of interest in epileptology, since impaired neuronal migration is one of the etiological factors in the development of TLE.

Neuronal migration is a complex process necessary for the correct layering of the cortex and the formation of functional neural networks in the brain. Previously, it was shown that three miRs (miR‒9, miR‒132, and miR‒137), which suffice in the brain, are involved in the regulation of neuronal migration [88]. miR‒9 and miR‒132 can also play the role of positive regulators of neuronal migration, preventing the expression of the forkhead box protein P2 (FOXP2) transcription factor [89]. In contrast, Franzoni et al. [86] showed that miR‒128 is a negative regulator of neuronal migration and that the onset of miR‒128 activity coincides with the cessation of upper neuronal migration. Manipulation of the time of miR‒128 expression can lead to suppression of migration processes and the formation of layers of the cortex. At least in part, this is accomplished through the regulation of the PHF6 transcriptional repressor via miR‒128. The authors also showed that the regulation of PHF6 by miR‒128 is important for two interdependent processes of maturation of neurons in the upper layer of the cortical lamina. miR‒128 and PHF6 interact in the regulation of dendritic branching of neurons in the upper layer of the cortex. Electrophysiological studies also demonstrate that the balance between miR‒128 and PHF6 affects the autonomic excitability of neurons. PHF6 knockdown has previously been shown to increase the excitability of heterotopic neurons that have been retained in the white matter of the brain due to impaired neuronal migration [90]. The cumulative effect of physiological changes caused by miR‒128 leads to an increase in neuronal excitability [85].

There is strong evidence for the presence of dendritic abnormalities in patients with TLE. These abnormalities and changes in the number of dendrites are observed in pyramidal neurons of the hippocampus, serrated granular cells in patients with mesial TLE, and in animals with a model of mTLE. On the other hand, it has been shown that miR‒134 is constitutively expressed in the bodies of neurons and dendrites of the adult brain [52]. It was revealed that overexpression of miR‒134 in neurons in vitro reduces the size of dendrites, while hypo-expression of miR‒134 leads to a slight increase in their size [50]. The mechanism of these changes was explained by the locally directed translation of the miR‒134 Lim domain containing kinase 1 (Limk1) inside the dendrites. Overexpression of miR‒134 in vivo using viral vectors resulted in a small but significant decrease in the branching of basal dendrites in pyramidal neurons in layer V of the cerebral cortex. Additionally, other targets for miR‒134 have been identified, including the RNA‒binding protein P53 upregulated modulator of apoptosis (Pum2), C-AMP response element-binding protein (CREB), and doublecortin (DCX). Thus, miR‒134 is a potentially important regulator of brain development and synaptic plasticity [91]. Changes in miR‒134 expression also led to changes in the number and size of dendritic spines on excitatory neurons, presumably through the target kinase LIM domain [92,93,94].

## 4. Discussion

miRs are short (approximately 22 nucleotides) RNA molecules that primarily act as antisense regulators of gene expression. The coordinating functions of miRs are important and described for each of the stages of anatomical and functional development of neurons in the cerebral cortex, from the processes of stem cell proliferation and neurogenesis, to neuronal growth and synaptogenesis, both during intrauterine development and in the postnatal period. The expression of active forms of miRs and their initial nuclear transcripts occurs via two processing events associated with the activity of RNA-ases. It is known that expression of miRs during brain development takes place at every stage of the biogenesis pathway [95,96,97].

The basic and clinical studies that we have analyzed demonstrate that changes in the expression of miRs at the level of the mesial regions of the temporal lobes of the brain and changes in the level of circulating miRs in blood plasma can be considered as potentially clinically significant diagnostic biomarkers of mTLE. Circulating miRs can also be considered as predictors of the development of therapeutic resistance to AEDs and a risk factor for the development of status epilepticus in patients with mTLE. Indeed, various types of miRs constantly circulate in the blood plasma in a stable form in patients with TLE; therefore, the determination of the expression level of diagnostically significant circulating miRs is promising for implementation in clinical practice.

The fundamental and clinical studies analyzed by us are shown in Table 1.

**Figure 2:**
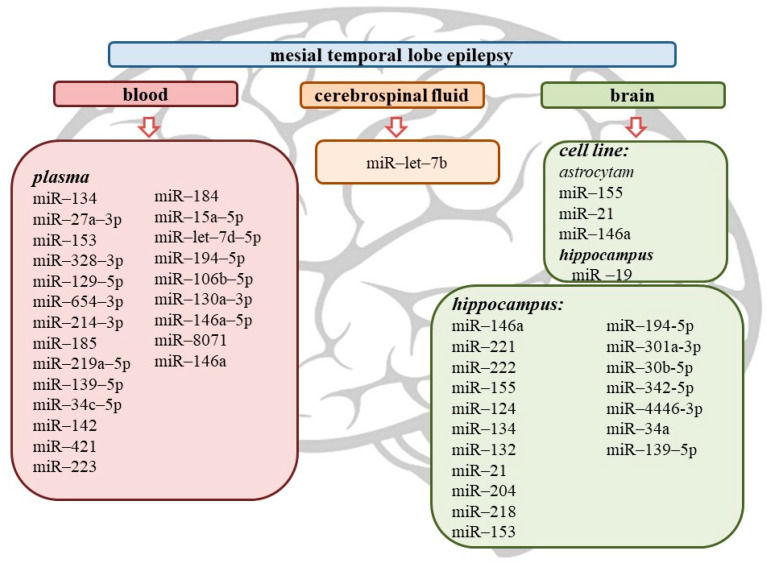
MicroRNA biomarkers for mesial temporal lobe epilepsy.

**Figure 3:**
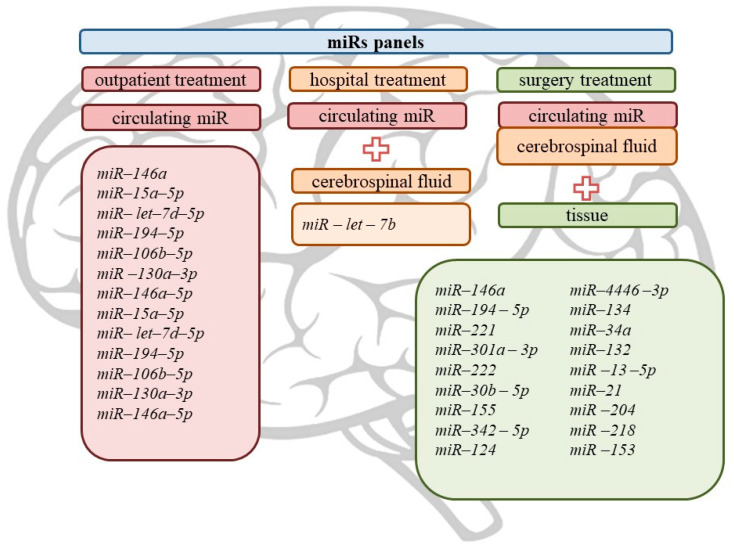
New microRNA diagnostic panels for mesial temporal lobe epilepsy.

The summary results of this review are presented in Figure 2 and Figure 3.

Overexpression of miR‒134 was also found in other experimental studies on an animal model of TLE and in the resected tissues of the temporal lobe of operated patients with therapeutically resistant mTLE. Studies in rodents have shown that hypoexpression of miR‒134 in the mesial regions of the temporal lobes can lead to a decrease in the epileptic threshold and reduce the risk of developing status epilepticus. In patients with TLE, the level of miR‒134 changes in the plasma suggests that miR‒134 may have diagnostic value as a biomarker of this disease [91].

For some miRs associated with the development of TLE, transcriptional mechanisms have been described. For example, the Mef2 protein is activated by neuronal activity and stimulates the expression of miR‒134 in neurons [98].

In studies on the validation of circulating miRs, which can be used as biomarkers of mesial TLE, miR–134 acts as the most promising diagnostic biomarker of this disease. Thus, the study by Avansini et al. [44] revealed statistically significant miR–134 hypoexpression in patients with mTLE in comparison with the control group (*p* < 0.001). The authors concluded that a decrease in miR–134 expression may be a potential additional diagnostic biomarker of mTLE. On the other hand, Jimenez-Mateos et al. [52] showed that overexpression of miR–134 occurred in areas of the hippocampus after seizures as a result of local post-convulsive neuronal damage. Overexpression of miR–134 was also observed in hippocampal neurons in mice with a mTLE model and in hippocampal tissue samples obtained by surgery from patients with therapeutically resistant mTLE. Although the functional significance of miR–134 in assessing changes in dendritic branching of hippocampal neurons is currently insufficiently understood, a temporary reduction in the number of dendritic spines leads to a decrease in NMDA-dependent signal transmission between neurons and protects hippocampal neurons from excitotoxic damage. In accordance with this hypothesis, on the example of an animal model of mTLE (mice) [99], when miR–134 expression did not change, the frequency of epileptic seizures and the risk of developing status epilepticus were shown to be 50% less than in animals with miR–134 overexpression at the hippocampal level [52]. Wang et al. [100] demonstrated overexpression of miR–134–5p in patients with new onset epilepsy. Blocking the expression of miR–134–5p in a variety of animal models of epilepsy reduced the duration of epileptic seizures and had a neuroprotective effect.

miR–153 is a conservative miR and at the same time is a candidate miR for assessing the regulation of teratogenesis and neurobehavioral disorders [101,102,103], which may be associated with mTLE. Additionally, miR–153 reduces the expression of HIF-1α in the brain of patients with therapeutically resistant TLE, lowering the resistance of neurons in the mesial regions of the temporal lobes of the brain to hypoxia and oxidative stress [104]. In turn, a decrease in the expression of HIF-1α increases the expression of P-glycoprotein, which leads to a change in the efflux of AED across the neuronal membrane and blood-brain barrier and increases the risk of developing therapeutic resistance to AEDs. On the other hand, miR–153 hypoexpression has been shown to be a risk factor for the development of TLE [103].

Research on candidate miRs is ongoing. At the same time, circulating miRs are of particular clinical interest, since blood samples from patients with mTLE are more accessible than samples of resected hippocampal tissue in clinical practice. This leads to an increase in the number of candidate miRs in mTLE (Table 2).

Huang et al. [105] described 42 exosomal circulating miRs in patients with mTLE-HS. At the same time, in mTLE, 25 miRs overexpression and 17 miRs hypoexpression were revealed. However, after validation of the obtained results, only overexpression of miR–129–5p, miR–214–3p, miR–219a–5p, and miR–34c–5p and hypoexpression of miR–421 and miR–184 were statistically significant. miR–184 showed the highest diagnostic value for the diagnosis of mTLE-HS with sensitivity of 88.9% and specificity of 83.3%. These six miRs are involved in the regulation of several candidate genes, encoding neurotrophin-, hippo-, p53-, TGF-beta, HIF-1-, and mTOR-related pathways. However, the limitation of this study is the small sample (36 patients, including 18 patients with mTLE-HS and 18 patients with mTLE without HS). A study by Yan S. et al. [45] showed that the miRs profile of plasma exosomes in patients with mTLE-HS differs in comparison with healthy controls, and miR–8071 hypoexpression has the best predictive value with sensitivity of 83.33% and specificity of 96.67%.

Raoof et al. [41] revealed overexpression of circulating miR–27a–3p, miR –328–3p, and miR–654–3p hypoexpression in plasma of patients with mTLE.

The question of the mechanisms of development of therapeutically resistant mTLE remains open at present. The role of miRs is one possible answer to this complex and multifaceted clinical question. However, the level of circulating miRs in plasma as a tool for assessing the fine cellular regulation of the development and functioning of neurons is subject to individual fluctuations and its assessment in clinical practice cannot always be unambiguous. However, the greatest interest in the diagnostic significance of circulating miRs is their role as biomarkers of therapeutic resistance in epilepsy. Thus, in a study by Wang et al. [47], hypoexpression of miR–194–5p, miR–301a–3p, miR–30b–5p, miR–342–5p, and miR–4446–3p was revealed in the group of patients with therapeutically resistant epilepsy compared with the group of patients sensitive to AEDs and the control group of healthy volunteers. The authors showed that miR–301a–3p hypoexpression has the best diagnostic value for therapeutically resistant epilepsy with sensitivity of 80.5% and specificity of 81.2%, and the level of its expression in plasma decreases depending on the severity of epileptic seizures. The diagnostic value of miR–301a–3p as a biomarker of therapeutic resistance to AEDs has been confirmed by other research [56,106]. It was found that the mechanism of the formation of therapeutic resistance to AED in the case of impaired expression of this miR is associated with an increase in the expression of nuclear factor kappa-light-chain-enhancer of activated B cells (NF-κB protein) [46].

Additionally, Wang et al. [107] revealed that changes in miRs expression are closely associated with the development of therapeutic resistance to AEDs, not only in patients with mTLE, but also in patients with other forms of epilepsy. For example, overexpression of miR–34a and miR–132 is associated with the death of brain neurons in the epileptogenic focus, which is caused by prolonged seizures, and pronounced miR–134 hypoexpression is associated with a low risk of epileptic seizures and status epilepticus. [108].

In a study by An et al. [109], a pronounced overexpression of miR–106b in blood plasma was found in patients with focal epilepsy (AUC = 0.786). Additionally, miR–106b overexpression was detected in patients with epilepsy in the study by Wang et al. [48]. However, these studies did not separately investigate miR–106b expression in patients with mTLE. miR–106b hypo-expression was detected in the study by McKiernan et al. [106] in patients with mTLE and in an animal model of epilepsy [110].

Genome-wide sequencing showed [46] that miR–185 was expressed differently in patients with therapeutic resistance to AEDs and those without therapeutic resistance.

miR–139–5p hypoexpression is associated with a significant increase in the expression level of the multidrug resistance protein (MRP1), which is a transporter of a wide range of AEDs. This was revealed in blood serum samples taken from children with therapeutically resistant epilepsy and in resected brain tissue samples from animal models of therapeutically resistant epilepsy [111]. miR–139–5p overexpression or MRP1 hypo-expression can reduce the risk of apoptosis and necrosis of neurons in the epileptogenic focus, as well as increase their sensitivity to AEDs [111].

Furthermore, suppression of miR–139–5p expression in hippocampal tissues modulates the NR2A-containing NMDA receptor during various phases of status epilepticus [112]. In an experiment on an animal model (newborn rats), miR–139–5p reduced the severity of damage to brain neurons due to epileptic seizures as a result of hypo-expression of protein dependent on human growth and transformation (HGTD-P) [113].

According to Benedittis et al. [114], pronounced overexpression of miR–142 and miR–223 was observed in patients with therapeutically resistant mTLE compared with those with benign disease. It is believed that the formation of therapeutic resistance to AEDs in patients with mTLE changes the expression of various genes and the activation of numerous transcription factors, which leads to a change in the structure of chromatin in accordance with its transcription or repression [115]. For this reason, among the main targets of miRs considered in this thematic review there is a signal transducer and activator of transcription 3 (STAT3), E2F transcription factor 1E (2F1), Sp1 transcription factor (SP1), HIF1a, and chromatin remodeling molecules (SWI/SNF related, matrix associated, actin dependent regulator of chromatin, subfamily d, member 1 (SMARCD1), coactivator associated arginine methyltransferase 1 (CARM1), and polypyrimidine tract binding protein 2 (PTBP2)).

Another important mechanism for the development of therapeutic resistance to AEDs is hyperactivation of phagocytosis by microglial cells [60], which leads to the launch of neurodegeneration processes and the loss of neurons in the area of the epileptic focus. Therefore, some inhibitors of cytokine signaling (for example, a suppressor of cytokine signaling 1–SOCS1), modulators of monocytes and neutrophils (for example, CXC motif chemokine ligand 2–CXCL2) of the polymer complex of inflammasome, are considered to be possible targets for miR–223 and miR–142 (for example, NLR family pyrin domain containing 3–NLRP3) and some factors are involved in the inflammatory process (CC motif chemokine ligand 3–CCL3), IL6). It has been hypothesized that the main role in the development of therapeutic resistance to AEDs is played by a group of transmembrane proteins that regulate the BBB permeability or the exchange of ions and small molecules between the brain and the extracerebral environment of the body (for example, Claudin1, cytochrome b5 type A–CYB5A, CF transmembrane conductance regulator—CFTR). Possibly, overexpression of miR–223 and miR–142 leads to changes in the permeability of the membranes of neurons and BBB endothelial cells, which, in turn, predisposes the development of therapeutic resistance to AED in patients with epilepsy. Interestingly, miR–223 expression is increased in patients with a later onset of epilepsy [114].

Four candidate genes have been identified that are involved in synaptic plasticity Rundabout Guidance Receptor 1 (*ROBO1*), Glutamate Metabotropic Receptor 1 (*GRM1*), Solute Carrier Family 1 Member 2 (*SLC1A2*), and G Protein Subunit Alpha I2 (*GNAI2*). The proteins encoded by these genes are regarded as targets for miR–218. It was shown that the expression of miR–218 and miR–204 was suppressed in hippocampal samples from patients with mTLE. A decrease in miR–218 expression in neurons of various parts of the hippocampus was reported in studies of an animal model of TLE in the latent and chronic phases of the disease. A noticeable loss of neurons revealed tissue samples of the mesial regions of the temporal lobes in mTLE-HS could mask the potential activation of this miR [54].

## 5. Limitations

The limitation of our systematic review is a 10-year period of publication analysis. In addition, the restriction is to search for publications only in English. Furthermore, we did not analyze preprints or conference materials (for example, posters).

An interesting question is the function of each of the miRs included in the analysis. We did not summarize the results of studies on each specific miR, not did we analyze the influence of the sex and age of the patients with mTLE on the level of miRs expression, since these differences were not studied in any of the publications analyzed by us.

The limitation of this review is the focus only on the impact of miRs on the effectiveness of AEDs in patients with epilepsy. However, it may be interesting to analyze studies of the effect of AEDs and other drugs on miRs‘ expression in patients with TLE.

These topics are important for discussion in future reviews.

## 6. Conclusions

Predicting the development and unfavorable course of mTLE in children and adults and assessing the risk of developing status epilepticus is an unsolved problem in modern epileptology. Along with this, it is important to further study the mechanisms of the development of therapeutic resistance of epileptic seizures to AEDs. Considering the high prevalence of TLE in children and adults in general and the high proportion of mTLE among other forms of focal epilepsy in particular, the study of the diagnostic and prognostic significance of circulating miRs in plasma as potential biomarkers is of undoubted importance for patients with mTLE. Investigation of the expression level of circulating miRs at various stages of disease development (debut, uncomplicated course, complicated course with epileptic status, and/or therapeutic resistance to AEDs) is of crucial clinical interest.

In the future, it will be possible to consider previously studied miRs, which have high specificity and sensitivity in mTLE, as prognostic biomarkers (predictors) of the risk of developing this disease in patients with potentially epileptogenic structural damage to the mesial regions of the temporal lobe of the brain (congenital disorders of the neuronal migration and neurogenesis, brain injury, neuro-inflammation, tumor, impaired blood supply, neurodegeneration, etc.).

However, it should be recognized that the translation of the results of basic studies of circulating miRs into clinical practice is difficult due to the ambiguous (contradictory) results of studies on the role of some miRs. This may be due to the different designs of the studies that we analyzed and the conduct of studies in different regions, with the inclusion of patients from different racial and ethnic groups. The level of miRs expression can be influenced not only by the onset and course of mesial TLE and the development of therapeutic resistance to AEDs, but also by the carriage of SNVs of the genes encoding these miRs.

## Figures and Tables

**Table 1:** MicroRNAs expression in plasma, cerebrospinal fluid, and brain tissues in patients with mesial temporal lobe epilepsy.

miR	Research Method	Tissue	Subjects	Effects	Authors, Year
Plasma studies of miRs in patients with mTLE					
3613–5p 4668‒5p 8071 197‒5p 4322 6781–5p	Case-control	Blood (plasma)	40 adults (mTLE‒HS). Mean age of men—26.21 years old. Mean age of women—28.32 years old.	miR–3613–5p overexpression is biomarkers of mTLE‒HS. miR–4668–5p, miR–8071, and/or miR–197–5p hypoexpression is a biomarker of mTLE‒HS. miR–8071 hypoexpession is biomarker seizures severity in mTLE.	Yan et al., 2017
129–5p 184 214–3p 219a–5p 34c–5p 421	Case-control	Blood (plasma)	Six adults (mTLE‒HS). Mean age—29 years old. 18 adults (mTLE). Mean age—27 years old.	miR–129–5p, miR–214–3p, miR–219a–5p, and and miR–34c–5p overexpression is potential biomarker for mTLE‒HS. miR–421 and miR–184 hypoexpression is potential biomarker of mTLE‒HS. Great diagnostic value of miR–184.	Huang et al., 2020
145 181c 199a 1183	Case-control	Blood (plasma)	20 adults (therapeutically resistant mTLE‒HS). Mean age—n/a.	miR–145, miR–181c, miR–199a, and miR –1183 overexpression is potential biomarkers of therapeutically resistant mTLE‒HS.	Antônio et al.,2019
134	Case-control	Blood (plasma)	79 adults (mTLE, including mTLE‒HS). Mean age—n/a.	miR–134 hypoexpression of may be a potential biomarker of mTLE (including mTLE‒HS).	Avansini et al., 2017
155	Case-control	Blood (plasma)	Adults (TLE, including mTLE‒HS). Mean age—72 years old.	miR–155 overexpression of is a biomarker for TLE (including mTLE‒HS).	Duan et al., 2018
155 0067835	Case-control	Blood (plasma)	22 adults (therapeutically resistant mTLE‒HS after anterior temporal lobectomy). Mean age—n/a.	miR–0067835 hypoexpression and miR–155 overexpression are biomarkers of high seizure rates and poor outcomes in surgery (anterotemporal lobectomy) for therapeutically resistant mTLE‒HS.	Gong et al., 2018
124	Case-control	Blood (plasma)	307 adults (TLE, including mTLE). Mean age—47.12 years old.	miR–124 expression did not change statistically significantly. The rs531564 miR–124 polymorphism did not differ in the study group and control and cannot be used as a TLE biomarker (in the Italian population).	Manna et al., 2016
146a	Case-control	Blood (plasma)	357 adults (TLE, including mTLE‒HS). Mean age—47.41 years old.	miR–146a expression of does not significantly affect the risk of developing TLE (including mTLE‒HS) and its severity.	Manna et al., 2013
27a–3p 328–3p 654–3p 24–3p 146a–5p 451a	Case-control	Blood (plasma)	102 adults (TLE, including 32 adults with therapeutically resistant TLE). Mean age of men—40.2 years old. Mean age of women—36.7 years old.	miR–27a–3p and miR–328–3p overexpression is a diagnostic biomarker for TLE. miR–654–3p hypoexpression of is a potential biomarker for TLE. The combination of the three miRs provides an additional discriminatory value.	Raoof et al., 2018
153	Case-control	Blood (plasma)	22 adults (therapeutically resistant mTLE). Mean age—n/a.	miR–153 hypoexpression of may be a biomarker for therapeutically resistant mTLE.	Gong et al., 2018
145 181c 199a 1183	Case-control	Blood (plasma)	20 adults (therapeutically resistant mTLE‒HS). Mean age—n/a.	miR–145, miR–181c, miR–199a, and miR–1183 overexpression is potential biomarkers of therapeutically resistant mTLE‒HS.	Antônio et al., 2019
153 543 194 494	Case-control	Blood (plasma)	88 adults (mTLE). Mean age—n/a.	miR–153 hypoexpression is a biomarker for therapeutically resistant mTLE.	Li et al., 2016
129–2–3p 935	Case-control	Blood (plasma)	25 adults (therapeutically resistant TLE). Mean age of men—55.68 years old. Mean age of women—55.77 years old.	miR–129–2–3p overexpression is a biomarker for high seizure rates and therapeutically resistant TLE.	Sun et al., 2016
143–3p 145–3p 365a–3p 532–5p 663b	Cross-sectional study	Blood (plasma)	15 adults (mTLE‒HS). Mean age—n/a.	miR–143–3p, miR–145–3p, miR–365a–3p and miR–532–5p overexpression is biomarker of recent seizure in patients with mTLE‒HS (found 30 min after seizure).	Surges et al., 2016
145–5p	Case-control	Blood (plasma)	40 adults (therapeutically resistant epilepsy, including 11 patients with mTLE‒HS). Mean age—28.5 years old.	miR–145–5p hypoexpression is a biomarker for TLE (including mTLE‒HS).	Shen et al., 2019
301a3p 194–5p 30b–5p 342–5p 4446–3p	Case-control	Blood (plasma)	Adults with mTLE who died from SUDEP. Mean age—23 years old.	miR–301a–3p overexpression of may be a potential biomarker for SUDEP in patients with TLE.	De Matteis et al., 2018
miR studies in resected tissue of patients with mTLE					
145 181c 199a 1183	Case-control	Resected tissue of the temporal lobe	20 adults (therapeutically resistant mTLE‒HS). Mean age—n/a.	miR–145, miR–181c, miR–199a, and miR–1183 overexpression is biomarker for therapeutically resistant mTLE‒HS.	Antônio et al., 2019
153 194 494 543	Case-control	Resected tissue of temporal lobe	88 adults (mTLE). Mean age—n/a.	miR–153 hypoexpression is a biomarker for therapeutically resistant mTLE.	Li et al., 2016
153	Case-control	Resected tissue of temporal lobe	22 adults (therapeutically resistant mTLE). Mean age—n/a.	miR–153 hypoexpression of may be a biomarker for therapeutically resistant mTLE.	Gong et al., 2018
30b–5p 194–5p 301a3p 342–5p 4446–3p	Case-control	Resected tissue of temporal lobe.	Adults with mTLE who died from SUDEP. Mean age—23 years old.	miR–301a–3p overexpression of may be a biomarker for mTLE.	De Matteis/ 2018
129–2–3p 935	Case-control	Resected tissue of temporal lobe	13 adults (therapeutically resistant TLE). Mean age of men—55.68 years old. Mean age of women—55.77 years old.	miR–129–2–3p overexpression is a biomarker for high seizure rates and therapeutically resistant TLE.	Sun et al., 2016
miR studies in the cerebrospinal fluid of patients with mTLE					
19b–3p 21–5p 451a 22–3p 23b–5p 34a–5p 124–3p 128–3p 132–3p 134–5p 146a–5p 155–5p 184 199a–5p 203a–3p 210–3p 219a–5p 324–5p	Case-control	Cerebro-spinal fluid	15 adults (TLE, including mTLE). Mean age of men—35.8 years old. Mean age of women—46.3 years old.	miR–19b–3p hypoexpression is a biomarker for TLE, including mTLE. miR–21–5p and miR–451 overexpression ay be new biomarker for TLE, including mTLE.	Raoof et al., 2017

**Table 2:** MicroRNAs are potential biomarkers for the development of therapeutic resistance to antiepileptic drugs in patients with mesial temporal lobe epilepsy.

miR	Research Method	Tissue	Subjects	Effects	Authors, Year
Studies in patients with epilepsy					
146a	Case-control	Resected hippocampal tissue	11 adults patients (6—mTLE-HS, 5—mTLE without HS). Mean age—31.5 and 35.1 years old, respectively.	miR–146a overexpression of is a diagnostic biomarker for therapeutically resistant mT LE.	Aronica et al., 2010
146a	Case-control	Resected hippocampal tissue	7 adults (mTLE-HS). Mean age—n/a.	miR–146a overexpression can increase IL-1β levels in chronic TLE by suppressing CFH (miR–146a-CFH-IL-1β loop).	Li et al., 2018
146a	Case-control	Resected hippocampal tissue	11 adults (mTLE without HS). Mean age—n/a.	miR–146a overexpression of may be a biomarker for mTLE.	Iyer et al., 2012
146a	Case-control	Deoxygenated blood	174 adults (mTLE). Mean age—26.33 years old.	miR–146a is biomarker for decrease in the incidence of epileptic seizures in patients with mTLE.	Cui et al., 2015
155	Case-control	Resected hippocampal tissue	8 children (mTLE-HS). Mean age—n/a.	miR‒155 overexpression of may be a biomarker for mTLE-HS. The TNF-α/miR‒155 axis may be a new target for antiepileptic therapy.	Ashhab et al., 2013
221 222	Case-control	Resected hippocampal tissue	21 adults (10—mTLE-HS, 11—mTLE without HS). Mean age—n/a.	miR‒221 and miR‒222 hypoexpression may be a biomarker for mTLE.	Kan et al., 2012
21 124 134 132	Case-control	Resected hippocampal tissue	5 adults (mTLE-HS). Mean age—n/a.	Overexpression of miR‒124 and miR‒134 may be a biomarker for mTLE-HS. miR‒132 and miR‒21 may be new targets for antiepileptic therapy.	Peng et al., 2013
15a–5p let–7d–5p 194–5p 106b–5p 130a–3p 146a–5p	Case-control	Deoxygenated blood	117 adults (mTLE). Mean age—29.8 years old.	Overexpression of miR–15a–5p, miR–let–7d–5p, miR–194–5p, miR–106b–5p, miR–130a–3p miR–146a–5p may be a biomarker for mTLE.	Wang et al., 2015
194–5p 301a–3p 30b–5p 342–5p 4446–3p	Case-control	Blood (plasma)	107 adults (therapeutically resistant epilepsy). Mean age—32.44 years old.	Hypoexpression of miR–194–5p, miR–301a–3p, miR–30b– 5p, miR–342–5p, miR–4446–3p may be a biomarker for therapeutically resistant epilepsy.	Wang et al., 2015
139–5p	Case-control	Blood (plasma)	26 children (therapeutically resistant epilepsy). Mean age—n/a.	miR–139–5p hypoexpression may be a biomarker for therapeutically resistant epilepsy.	Wang et al., 2020
204 218	Case-control	Resected hippocampal tissue	20 adults (15—mTLE-HS, 5—mTLE without HS). Mean age—40.5 and 45.7 years old, respectively.	Hypoexpression of miR–218 and miR–204 may be a biomarker for mTLE-HS.	Kaalund et al., 2014
153	Case-control	Blood (plasma). Resected tissue of the temporal lobe	22 adults (therapeutically resistant mTLE). Mean age—n/a.	miR–153 overexpression of may be a biomarker for therapeutically resistant mTLE.	Gong et al., 2018
129–2–3p 935	Case-control	Resected tissue of the temporal lobe	13 adults (therapeutically resistant TLE). Mean age of men—55.68 years old. Mean age of women—55.77 years old.	miR–129–2–3p overexpression of is a biomarker for high seizure rates and therapeutic resistance to AEDs in TLE patients.	Sun et al., 2016
142 146a 223 138-5p 298	Case-control	Blood (plasma)	10 adults (therapeutically resistant TLE). Mean age—43.65 years old.	Overexpression of miR–223 and miR –142 may be a biomarker for therapeutically resistant TLE.	Benedittis et al., 2021
Animal model studies of epilepsy					
146a	Experimental (electrical model of epilepsy)	Resected hippocampal tissue	Adult male Sprague-Dawley rats (weight 300–500 g). Mean age—n/a.	miR–146a overexpression of is a biomarker for TLE.	Aronica et al., 2010
139–5p	Experimental (electrical model of epilepsy)	Resected brain tissue	30 adult male Sprague-Dawley rats (weight 180–220 g). Mean age—n/a.	miR–139–5p hypo-expression of may be a biomarker for therapeutically resistant epilepsy.	Wang et al., 2020
let–7b	Experimental model of epilepsy	Resected brain and spinal cord tissue Cerebrospinal fluid	Mice (C57Bl/6J (wild type); Tlr7 -/- and Myd88 -/-; CD11b-HSVTK). Mean age—n/a.	miR–let–7b overexpression is observed in epileptic seizures and stimulates TLR7, thereby initiating neuronal apoptosis in the epileptogenic focus area.	Lehmann et al., 2012
146a	Experimental (cain model of epilepsy)	Resected hippocampal tissue	Adult male Sprague-Dawley rats (weight 250–280 g). Mean age—n/a.	miR–146a overexpression of can increase IL-1β levels in therapeutically resistant TLE by suppressing CFH (miR–146a–CFH–IL–1β loop). Modulation of the miR–146a–CFH–IL–1β loop may be a new therapeutic target for mTLE.	Li et al., 2018
21 124 132 134	Experimental (pilocartpin model of epilepsy)	Resected hippocampal tissue	18 male Sprague-Dawley rats. Mean age—25 days.	Overexpression of miR124 and miR–134 may be a biomarker of mTLE. miR–132 and miR–21 may be new targets for antiepileptic therapy.	Peng et al., 2013
132	Experimental (cain model of epilepsy)	Resected hippocampal tissue	Adult male mice C57BL/6 (weight 20–22 g) Mean age—n/a.	miR‒132 hypo-expression occurs after epileptic seizure. miR–132 have neuroprotective effect.	Jimenez-Mateos et al., 2011
34a	Experimental (cain model of epilepsy)	Resected hippocampal tissue	Adult male mice C57BL/6 (weight 20–22 g). Mean age—n/a.	miR–34a expression is not significant for neuronal death caused by epileptic seizures.	Sano et al., 2012
128b	Experimental model of epilepsy	Resected brain tissue	Adult mice C57/Bl6J. Mean age—n/a.	miR–128b overexpression disrupts the stability of several target genes is associated with plasticity and regulates the formation of fear extinction memory.	Lin et al., 2011

## Data Availability

Not applicable.
